# Analysis of antibiotic resistance phenotypes and genes of *Escherichia coli* from healthy swine in Guizhou, China

**DOI:** 10.4102/ojvr.v88i1.1880

**Published:** 2021-02-24

**Authors:** Bo Yu, Yanan Zhang, Li Yang, Jinge Xu, Shijin Bu

**Affiliations:** 1 College of Veterinary Medicine, Yangzhou University, Yangzhou, China; 2 Institute of Animal Husbandry and Veterinary Medicine, Guizhou Academy of Agricultural Sciences, Guiyang, China

**Keywords:** antimicrobial resistance, extended-spectrum β-lactamase, ESBLs, *E. coli* from swine, plasmid-mediated quinolone resistance, PMQR, resistance genes

## Abstract

This study was carried out to investigate the resistance phenotypes and resistance genes of *Escherichia coli* from swine in Guizhou, China. A total of 47 *E. coli* strains isolated between 2013 and 2018 were tested using the Kirby–Bauer (K–B) method to verify their resistance to 19 common clinical antimicrobials. Five classes consisting of 29 resistance genes were detected using polymerase chain reaction. The status regarding extended-spectrum β-lactamase (ESBL) and the relationship between ESBL CTX-M-type β-lactamase genes and plasmid-mediated quinolone resistance (PMQR) genes were analysed. A total of 46 strains (97.9%) were found to be multidrug resistant. Amongst them, 27 strains (57.4%) were resistant to more than eight antimicrobials, and the maximum number of resistant antimicrobial agents was 16. Twenty antibiotic resistance genes were detected, including six β-lactamase genes blaTEM (74.5%), blaCTX-M-9G (29.8%), blaDHA (17.0%), blaCTX-M-1G (10.6%), blaSHV (8.5%), blaOXA (2.1%), five aminoglycoside-modifying enzyme genes aac(3′)-IV (93.6%), aadA1 (78.7%), aadA2 (76.6%), aac(3′)-II c (55.3%), aac(6′)-Ib (2.1%) and five amphenicol resistance genes floR (70.2%), cmlA (53.2%), cat2 (10.6%), cat1 (6.4%), cmlB (2.1%), three PMQR genes qnrS (55.3%), oqxA (53.2%), qepA (27.7%) and polypeptide resistance gene mcr-1 (40.4%). The detection rate of ESBL-positive strains was 80.9% (38/47) and ESBL TEM-type was the most abundant ESBLs. The percentage of the PMQR gene in blaCTX-M-positive strains was high, and the detection rate of blaCTX-M-9G was the highest in CTX-M type. It is clear that multiple drug resistant *E. coli* is common in healthy swine in this study. Extended-spectrum β-lactamase is very abundant in the *E. coli* strains isolated from swine and most of them are multiple compound genotypes.

## Background

Colibacillosis is a common infectious disease in swine caused by pathogenic *Escherichia coli*, and the main manifestations are sepsis, yellow scour and white scour in piglets. As there are many *E. coli* serotypes, vaccine-based control is not effective and antimicrobials are the main treatment methods at present (Yan & Zhao 2018; Zang & Zhou 2019). With high-dose and wide use of antimicrobials in breeding factories and livestock farms, the multidrug resistant (MDR) problem of bacteria is becoming increasingly serious. Especially, the infection caused by extended-spectrum β-lactamase (ESBL) producing bacteria increases the difficulty to control and represents a major global public health concern. *Escherichia coli* with CTX-Ms are the most common species associated with global ESBLs (Peirano & Pitout 2019). Currently, CTX-M-15 (forming part of the CTX-M-1 subfamily first described in *E. coli* in 2001 from India [Karim et al. 2001]) is the most frequent CTX-M worldwide. This is closely followed by CTX-M-14 (forming part of the CTX-M-9 subfamily first reported in *E. coli, K. pneumoniae* and *Shigella* spp. isolated in Korea in 2001 [Pai et al. 2001]). In the meantime, antibiotic resistance genes (ARGs) carried by *E. coli* of food animal origin would spread along the food chain and eventually cause harm to the public and human health (Gao, Han & Guan 2017; Yin, Chen & Cao 2018). Therefore, it is necessary to monitor the prevalence of drug resistance and ARGs in animals, particularly in swine.

This study investigated the prevalence of resistance phenotypes and resistance genes of *E. coli* isolated from swine in Guizhou in recent years, analysed ESBL status and the relationship between CTX-M-type β-lactamase genes and plasmid-mediated quinolone resistance (PMQR) genes. The findings would provide cues for rationale use of antimicrobials in veterinary practice to reduce transmission of drug resistance in the animal.

## Materials and methods

### *Escherichia coli* strains

A total of 112 faecal swab samples were collected from 47 large-scale pig farms in 13 counties and cities, including Guiyang, Anshun, Bijie, Qianxinan, Qiandongnan and Qiannan in Guizhou Province between 2013 and 2018. The samples were taken from diseased piglets with yellow and white scour symptoms, streaked out on Mackay medium and cultured at 37 °C for 24 h. Pink colonies were picked and purified on Luria-Bertani (LB) agar plate at 37 °C for 24 h, and the plates were stored at 4 °C for subsequent use. Isolated bacterial strains were cultured in LB medium at 37 °C for 24 h and tested according to Bergey’s Manual of Systematic Bacteriology. The strains were further classified using 16s ribosomal ribonucleic acid (rRNA) sequences, which were sequenced using a pair of universal primers (16S-F, 5′-TGT GGG AAC GGC GAG TCG GAA TAC-3′; 16S-R, 5′-GGG CGC AGG GGA TGA AAC TCA AC-3′) (Shahi, Singh & Kumar 2013).

### Reagent and culture medium

MacConkey agar medium, eosin-methylene blue agar medium, LB broth, LB agar medium, casein hydrolysate agar (MH agar, Muller-Hinton agar), and casein hydrolysate broth (MH broth, Muller-Hinton broth) were purchased from Huankai Microbial Technology, Guangzhou; DNA markers and Premix Taq (EX Taq version 2.0 plus dye) were purchased from Takara Bio, Dalian; agarose was purchased from Biowest, Spain. Antimicrobial susceptibility disks were purchased from Binhe Microorganism Reagent, Hangzhou and they were used to test susceptibility to beta-lactam antibiotics beta-lactam antibiotics (cefotaxime 30 micrograms [*μ*g], ceftazidime 30 *μ*g, cefoxitin 30 *μ*g, ampicillin 10 *μ*g, amoxicillin-clavulanate 20 *μ*g and 10 *μ*g), aminoglycosides (amikacin 30 *μ*g, gentamicin 10 *μ*g, kanamycin 30 *μ*g, streptomycin 10 *μ*g, neomycin 30 *μ*g), tetracyclines (tetracycline 30 *μ*g, doxycycline 30 *μ*g), amphenicols (chloramphenicol 30 *μ*g, florfenicol 30 *μ*g), fluoroquinolones (ciprofloxacin 5 *µ*g, enrofloxacin 5 *μ*g, levofloxacin 5 *μ*g), polymyxin B (300 units) and sulfisoxazole (250 *μ*g and 300 *μ*g).

### Drug susceptibility test

The Kirby–Bauer method (K–B method) was used to test the susceptibility to antimicrobials. *Escherichia coli* ATCC^®^25922 was used as the quality control strain in antimicrobial susceptibility testing. The breakpoints for tested drugs were used as recommended by the standards and guidelines described by the CLSI (2018b: M100) and veterinary CLSI (VET01-A4/VET01-S2) (CLSI 2018a, 2018b). Based on the test, *E. coli* was classified as susceptible (S), intermediate (I) and resistant (R).

### Test of antibiotic resistance genes

#### Extraction of bacterial deoxyribonucleic acid

Strains stored at −80 °C were inoculated in LB broth for recovery, streaked on eosin-methylene blue agar medium and incubated at 37 °C overnight. Fresh single colonies on the eosin-methylene blue plate were inoculated in 3 mL LB broth and cultured at 37 °C on a shaker operated at 200 revolutions per minute (r/min) for 12 h. The culture was then centrifuged at 12 000 r/min for 5 min. Bacterial pellet was suspended in 500 *μ*L 1 × TE, vortexed and boiled for 10 min. Bacterial DNA was extracted using TIANamp Bacterial DNA Extraction Kit (TIANGEN, China) according to manufacturer’s instruction.

#### Polymerase chain reaction

Polymerase chain reaction (PCR) was used to amplify genes from the *E. coli* strains, including β-lactamase genes (*bla*_DHA_, *bla*_CMY-2_, *bla*_TEM_, *bla*_SHV_, *bla*_CTX-M-1G_, *bla*_CTX-M-9G_, *bla*_OXA_), aminoglycoside modifying enzyme genes (*aac*(*3’*)*-Ia, aac*(*3’*)*-IIc, aac*(*3’*)*-IV, aac*(*6’*)*-Ib, aadA1, aadA2, rmtA, rmtB*), amphenicol resistance genes (*cat1, cat2, cmlA, cmlB, floR*), PMQR genes (*qnrA, qnrB, qnrC, qnrD, qnrS, qepA, oqxA, oqxB*) and polypeptide resistance gene *mcr-1*. The primers for PCR are listed in Table 2 and were synthesised at Sangon Biotech, Shanghai.

Polymerase chain reaction amplification was performed in a final volume of 25 *μ*L containing 1 *μ*L bacterial DNA, 0.5 *μ*L each of primers, 12.5 *μ*L *Premix Taq* (*EX Taq* version 2.0 plus dye). Parameters for PCR were: initial denaturation for 10 min at 94 °C, followed by 30 cycles of 45 s at 94 °C, 45 s at annealing temperature and 1 min at 72 °C with a final extension of 10 min at 72 °C. The annealing temperatures for each pair of primers are listed in Table 1. The amplified PCR products were separated by electrophoresis on a 1% agarose gel in Tris-borate-Ethylenediaminetetraacetic acid (EDTA) buffer (TBE) containing 1% Goldview Nucleic Acid Gel Stain (Solarbio, Beijing, China) and monitored in gel documentation unit (BioRad Laboratories, United States). The amplicons were sequenced at Sangon Biotech, Shanghai. Sequence alignments were carried out using BLAST on NCBI website http://www.ncbi.nlm.nih.gov/ to determine the genes of interest.

**TABLE 1:** Primers for polymerase chain reaction.

Genes	Primer sequence (5′-3′)	Product size (bp)	Annealing temperature (°C)	Reference
*bla*_DHA_	F: AACTTTCACAGGTGTGCTGT	387	56	Pai, Seo & Choi, 2007
	R: CCGTACGCATACTGGCTTTC			
*bla*_CMY-2_	F: ATGATGAAAAAATCGTTATGC	1143	55	Yan et al. 2007
	R: TTGCAGCTTTTCAAGAATGCG			
*bla*_TEM_	F: ATAAAATTCTTGAAGACGAAA	1080	52	Weill et al. 2004
	R: GACAGTTACCAATGCTTAATC			
*bla*_SHV_	F: CACTCAAGGATGTATTGTG	885	55	Brinas et al. 2005
	R: TTAGCGTTGCCAGTGCTCG			
*bla*_CTX-M-1G_	F: CTTCCAGAATAAGGAATCCC	949	55	Liu et al. 2007
	R: CGTCTAAGGCGATAAACAAA			
*bla*_CTX-M-9G_	F: TGACCGTATTGGGAGTTTG	902	58.5	Liu et al. 2007
	R: ACCAGTTACAGCCCTTCG			
bla_OXA_	F: ATATCTCTACTGTTGCATCTCC	619	48	Colom et al. 2003
	R: AAACCCTTCAAACCATCC			
aac(3′)-Ia	F: TTACGCAGCAGCAACGATGT	402	58.5	Sun et al. 2012
	R: GTTGGCCTCATGCTTGAGGA			
aac(3′)-IIc	F: AACCGGTGACCTATTGATGG	774	58.5	Sun et al. 2012
	R: TGTGCTGGCACGATCGGAGT			
aac(3′)-IV	F: GGCCACTTGGACTGATCGAG	609	58.5	Sun et al. 2012
	R: GCGGATGCAGGAAGATCAAC			
aac(6′)-Ib	F: TTGCGATGCTCTATGAGTGGCTA	482	55	Park et al. 2006
	R: CTCGAATGCCTGGCGTGTTT			
aadA1	F: AGGTAGTTGGCGTCATCGAG	589	58.5	Sun et al. 2012
	R: CAGTCGGCAGCGACATCCTT			
aadA2	F: GGTGCTAAGCGTCATTGAGC	470	51	Sun et al. 2012
	R: GCTTCAAGGTTTCCCTCAGC			
rmtA	F: CTAGCGTCCATCCTTTCCTC	635	55	Chen et al. 2004
	R: TTGCTTCCATGCCCTTGCC			
rmtB	F: ACATCAACGATGCCCTCAC	724	54	Chen et al. 2004
	R: AAGTTCTGTTCCGATGGTC			
cat1	F: CTTGTCGCCTTGCGTATAAT	508	54	Chen et al. 2004
	R: ATCCCAATGGCATCGTAAAG			
cat2	F: AACGGCAYGATGAACCTGAA	547	50	Chen et al. 2004
	R: ATCCCAATGGCATCGTAAAG			
cmlA	F: CGCCACGGTGTTGTTGTTAT	394	55	Chen et al. 2004
	R: GCGACCTGCGTAAATGTCAC			
cmlB	F: ACTCGGCATGGACATGTACT	840	55	Chen et al. 2004
	R: ACGGACTGCGGA ATCCATAG			
floR	F: CTGAGGGTGTCGTCATCTAC	673	58	Chen et al. 2004
	R: GCTCCGACAATGCTGACTAT			
qnrA	F: ATTTCTCACGCCAGGATTTG	516	58	Robicsek et al. 2006
	R: GATCGGCAAAGGTTAGGTCA			
qnrB	F: GATCGTGAAAGCCAGAAAGG	469	58	Robicsek et al. 2006
	R: ACGATGCCTGGTAGTTGTCC			
qnrC	F: ATTTCTCACAGGCAAACT	666	53	Sun et al. 2012
	R: CTGGAATAACAATCACCC			
qnrD	F: TTTTCGCTAACTAACTCGC	984	54.4	Sun et al. 2012
	R: GAAAGGATAAACAGGCAAAT			
qnrS	F: ACGACATTCGTCAACTGCAA	417	56	Robicsek et al. 2006
	R: TAAATTGGCACCCTGTAGGC			
qepA	F: GCAGGTCCAGCAGCGGGTAG	199	60	Yamane et al. 2008
	R: CTTCCTGCCCGAGTATCGTG			
oqxA	F: GATCAGTCAG TGGGATAGTTT	670	52	Hansen et al. 2005
	R: TACTCGGCGTTAACTGATTA			
oqxB	F: TTCTCCCCCGGCGGGAAGTAC	512	52	Kim et al. 2009
	R: CTCGGCCATTTTGGCGCGTA			
mcr-1	F: CGGTCAGTCCGTTTGTTC	309	52.5	Liu et al. 2016
	R: CTTGGTCGGTCTGTAGGG			

bp, base pairs.

### Ethical consideration

No animal-related tests were involved in this study, and the faecal swab samples were provided by the farm owners who volunteered to participate in the study. The sampling protocols, strain isolation and experiments were conducted in accordance with Section 20 of the *Animal Diseases Act* of 1984 (Act No. 35 of 1984), Technical Guidelines for Isolation and Identification of Animal Origin *Escherichia coli* (DB51/T 2084–2015) and the Declaration of Helsinki, and were approved by the Animal Research Ethics Committee of Institute of Animal Husbandry and Veterinary Medicine, the College of Veterinary Medicine, Yangzhou University, Yangzhou, China.

## Results

### Isolated *Escherichia coli* strains were multidrug resistant

Drug susceptibility tests showed that 47 *E. coli* strains isolated from swine had the highest rates of resistance to tetracycline and doxycycline, which were both 95.7% (*n* = 45), followed

by resistance to sulfaisoxazole (89.4%, *n* = 42), ceftazidime (70.2%, *n* = 33), amoxicillin (63.8%, *n* = 30), chloramphenicol (63.8%, *n* = 30), ampicillin (61.7%, *n* = 29), florfenicol (57.4%, *n* = 27) and kanamycin (53.2%, *n* = 25). The rates of resistance to enrofloxacin, gentamicin, polymyxin B and cefotaxime were 46.8% (*n* = 22), 40.4% (*n* = 19), 38.3% (*n* = 18) and 31.9% (*n* = 15), respectively. The strains had relatively lower rates of resistance to ciprofloxacin, levofloxacin, neomycin and streptomycin, which were 29.8% (*n* = 14), 29.8% (*n* = 14), 19.1% (*n* = 9) and 17.0% (*n* = 8), respectively. In addition, the tests also showed that the strain had a rate of resistance to cefoxitin at only 8.5% (*n* = 4), and none of them was resistant to amikacin (Table 2).

**TABLE 2:** Antibiotic resistance of 47 *Escherichia coli* isolates over different years.

Antimicrobial agents	Resistant		Years					
			2013–2014 (*n* = 15)		2015–2017 (*n* = 18)		2018 (*n* = 14)	
	No. resistant strain	%	No. resistant strain	%	No. resistant strain	%	No. resistant strain	%
Cefotaxime	15	31.9	4	26.7	8	44.4	3	21.4
Ceftazidime	33	70.2	10	66.7	12	66.7	11	78.6
Cefoxitin	4	8.5	1	6.7	1	5.6	2	14.3
Ampicillin	29	61.7	8	53.3	10	55.6	11	78.6
Amoxicillin-clavulanate	30	63.8	8	53.3	11	61.1	11	78.6
Amikacin	0	0.0	0	0.0	0	0.0	0	0.0
Gentamicin	19	40.4	6	40.0	10	55.6	3	21.4
Kanamycin	25	53.2	11	73.3	8	44.4	6	42.9
Streptomycin	8	17.0	2	13.3	4	22.2	2	14.3
Neomycin	9	19.1	2	13.3	4	22.2	3	21.4
Ciprofloxacin	14	29.8	4	26.7	6	33.3	4	28.6
Enrofloxacin	22	46.8	5	33.3	8	44.4	9	64.3
Levofloxacin	14	29.8	4	26.7	5	27.8	5	35.7
Florfenicol	27	57.4	8	53.3	11	61.1	8	57.1
Chloramphenicol	30	63.8	10	66.7	10	55.6	10	71.4
Tetracycline	45	95.7	15	100.0	16	88.9	14	100.0
Doxycycline	45	95.7	14	93.3	17	94.4	14	100.0
Polymyxin B	18	38.3	6	40.0	10	55.6	2	14.3
Sulfisoxazole	42	89.4	14	93.3	15	83.3	13	92.9

When analysed based on isolation time (Table 2), the rates of resistance to ampicillin, amoxicillin, enrofloxacin and levofloxacin increased from 2013 to 2018, whilst the rate of resistance to kanamycin decreased during the same period. On other hand, the rates of resistance to ceftazidime and cefoxitin remained unchanged from 2013 to 2017, and increased in 2018; the rates of resistance to cefotaxime, gentamicin, streptomycin and neomycin were the highest from 2015 to 2017, and decreased in 2018. The rates of resistance of the 47 strains to florfenicol, chloramphenicol, tetracycline, doxycycline and sulfaisoxazole were higher than 50% from 2013 to 2018. For polymyxin B, the rate of resistance was 14.3% (2/14) in 2018, obviously lower than that from 2013 to 2014 (12.8%) and from 2015 to 2017 (55.6%, 10/18).

**FIGURE 1:**
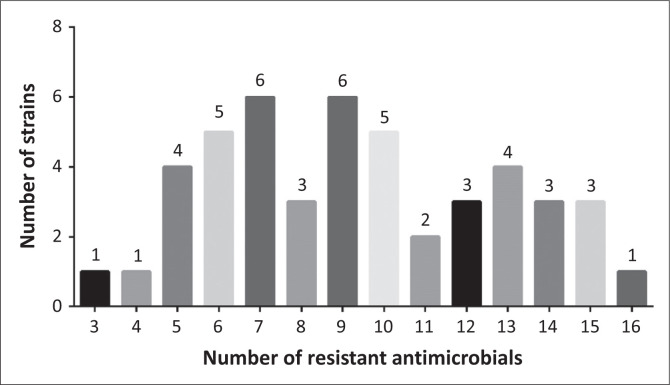
Distribution of *Escherichia coil* strains resistant to antimicrobials.

According to the criteria of multidrug resistance test, all 46 strains tested were resistant to more than three classes of antimicrobial agents, indicating serious multidrug resistance in these strains (Figure 1). Amongst them, 27 strains were resistant to more than eight antimicrobials, four strains were resistant to 15 antimicrobials, of which, ECP27 resisted to as many as 16 antimicrobials (Supplementary Table 1).

### Antibiotic resistance genes were detected in all isolates

In this study, 20 ARGs were detected (Table 3) using PCR-based assays and verified by sequencing and sequence alignment with BLASL. They were generally consistent with respective resistance phenotypes. Of which, *aac*(*3*′)*-IV* (93.6%, *n* = 44), *aadA1* (78.7%, *n* = 37), *aadA2* (76.6%, *n* = 36), *bla*_TEM_ (74.5%, *n* = 35), *floR* (70.2%, *n* = 33), *qnrS* (55.3%, *n* = 26), *aac(3*′*)-IIc* (55.3%, *n* = 26), *cmlA* (53.2%, *n* = 25) and *oqxA* (53.2%, *n* = 25) were prevalent with higher detection rates. Amongst the 47 *E. coli* strains, 39 strains carried β-lactamase genes with a detection rate of 83.0%, of which, the detection rates of *bla*_TEM_, *bla*_DHA_, *bla*_SHV_ and *bla*_OXA_ were 74.5% (35/47), 17.0% (8/47), 8.5% (4/47) and 2.1% (1/47), respectively. The *bla*_CMY-2_ gene was not detected, the detection rate of *bla*_CTX-M-9G_ in CTX-M was 29.8% (highest) and the detection rate of *bla*_CTX-M-1G_ was 10.6%. Amongst the strains positive for aminoglycoside-modifying enzyme genes, only one strain was positive for *aac*(*6*′)*-Ib*. The detection rates of *cmlA* (53.2%, 25/47) and *floR* (70.2%, 33/47) were higher, which were the main causes for amphenicols resistance. Three PMQR genes *qnrS, oqxA* and *qepA* were detected at rates of 55.3% (26/47), 53.2%, (25/47) and 27.7% (13/47), respectively. In addition, the detection rate of the mobilised colistin resistance gene *mcr-1* was 40.4%.

**TABLE 3:** Detection rate of antibiotic resistance genes in 47 *Escherichia coli* isolates.

Antibiotic resistance genes	Detection rate	
	No. positive strain	%
**β-lactamase**		
*bla*_DHA_	8	17.0
*bla*_TEM_	35	74.5
*bla*_SHV_	4	8.5
*bla*_CTX-M-1G_	5	10.6
*bla*_CTX-M-9G_	14	29.8
*bla*_OXA_	1	2.1
**Aminoglycoside-modifying enzyme gene**		
*aac(3′)-IIc*	26	55.3
*aac(3′)-IV*	44	93.6
*aac(6′)-Ib*	1	2.1
*aadA1*	37	78.7
*aadA2*	36	76.6
**Amphenicol resistance genes**		
*cat1*	3	6.4
*cat2*	5	10.6
*cmlA*	25	53.2
*cmlB*	1	2.1
*floR*	33	70.2
**PMQR**		
*qnrS*	26	55.3
*qepA*	13	27.7
*oqxA*	25	53.2
**MobiliSed colistin resistance gene**		
*mcr-1*	19	40.4

PMQR, plasmid-mediated quinolone resistance.

Antibiotic resistance genes were detected in all 47 strains, of which, 28 (59.6%) strains had more than eight ARGs, nine (19.1%) strains had more than 10 ARGs, and ECP27 had 14 ARGs (Supplementary Table 1).

### Various extended-spectrum β-lactamase genes were present in the isolates

**FIGURE 2:**
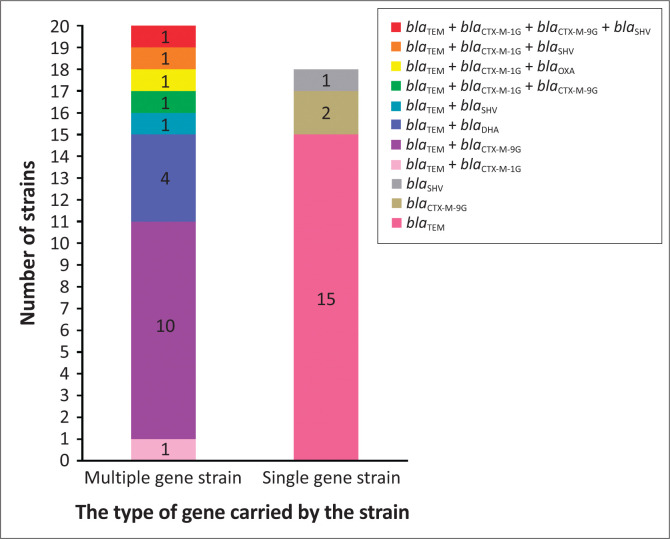
Genetic compositions of extended-spectrum β-lactamase strains. Left column: multiple gene strains, Right column: single gene strains.

Amongst the 47 strains tested, 38 (80.9%) strains carried *ESBLs* and 20 of them harbored various number of *ESBLs*. The most frequent was *bla*_TEM_+*bla*_CTX-M-9G_ (26.3%, 10/38); three strains were found to have three *ESBLs* genes and ECP27 had four *ESBLs* genes *bla*_TEM_, *bla*_CTX-M-1G_, *bla*_CTX-M-9G_ and *bla*_SHV_ (Figure 2, Supplementary Table 1).

### Various plasmid-mediated quinolone resistance genes were detected in CTX-M positive strains

**FIGURE 3:**
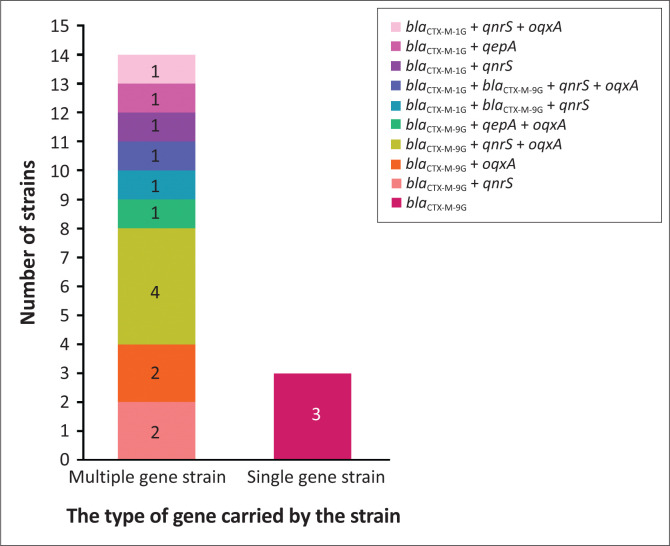
Genetic compositions of CTX-M positive strains. Left column: multiple gene strains, Right column: single gene strains.

We analysed the PMQR genes in 17 strains carrying *bla*_CTX-M_ and the results are shown in Figure 3. The PMQR genes were found in 14 strains, including strains with two genes *bla*_CTX-M-9G_+*qnrS* (two strains), *bla*_CTX-M-9G_+*oqxA* (two strains), *bla*_CTX-M-1G_+*qnrS* (one strain) and *bla*_CTX-M-1G_+*qepA* (one strain), three genes *bla*_CTX-M-9G_+*qnrS*+*oqxA* (four strains), *bla*_CTX-M-9G_+*qepA*+*oqxA* (one strain), *bla*_CTX-M-1G_+*bla*_CTX-M-9G_+*qnrS* (one strain) and *bla*_CTX-M-1G_+*qnrS*+*oqxA* (one strain) and four genes *bla*_CTX-M-1G_+*bla*_CTX-M__-9G_+*qnrS*+*oqxA* (one strain), of which, *bla*_CTX-M-9G_+*qnrS*+*oqxA* were the most frequent (23.5%, 4/17).

## Discussion

We investigated 47 *E. coli* strains isolated from swine in Guizhou from 2013 to 2018 for their resistance to 21 common antimicrobial agents. The results showed serious multidrug resistance in this region. The isolated strains have the highest resistance to the tetracycline antimicrobials, which are comparable to the rates of resistance to tetracycline (97.6%) and doxycycline (89.3%) in *E. coli* from swine in Guizhou in 2018 (Wang & Tan 2018). The rate of resistance to sulfaisoxazole is 89.4%, which is close to the rate of resistance to sulfonamide antimicrobials in 2018 (Kou, Liu & Tan 2018). The results may be related to the long-term irrational use of these three antibiotics in the prevention and treatment of piglet respiratory diseases and bacterial diarrhoea in pig breeding in Guizhou.

The strains are also resistant to β-lactam drugs, the rates of resistance to third-generation cephalosporin ceftazidime and cefotaxime were 70.2% and 31.9%, respectively, which were higher than that of second-generation cephalosporin drug cefoxitin (8.5%). This may be attributed to the irrational use of third-generation cephalosporins and the less use of second-generation cephalosporins in the pig farms in Guizhou in recent years.

For aminoglycoside resistance, the highest rates were resistant to kanamycin and gentamicin, followed by neomycin and streptomycin. However, none of the strain is resistant to amikacin. These results are consistent with earlier reports for Guangxi (Ge, Shi & Hu 2019), Chongqing (Chen & Liao 2019), Fujian (Yang, Luo & Xie 2018) and southeastern Shanxi (Shen, Kong & Guo 2019). This could be because of the less use of amikacin in the pig farms for disease treatment and prevention in Guizhou. The rates of resistance to aminoglycoside gentamicin, streptomycin, ciprofloxacin (a fluoroquinolone antimicrobial agent) and polymyxin (a polypeptide antimicrobial agent) are obviously lower than the rates of resistance (gentamicin 81.10%, streptomycin 53.66%, ciprofloxacin 53.05% and polymyxin 51.83%) observed in Guizhou in 2016 (Cao, Tan & Liu 2016), whilst the rate of resistance to florfenicol (an amphenicol agent) is obviously higher than previously reported rate (Cao et al. 2016). It is likely that the irrational use of fluorophenicol in the pig farms in recent years and the less use of gentamicin, streptomycin and polymyxins are responsible for the situation. At the same time, the reduction in ciprofloxacin resistance rate may be related to China’s ban on the use of four antibiotics in aquaculture from 2016, as well as improved management of livestock form and changes of antimicrobials used and administration method of antimicrobials.

From 2 to 14 ARGs are detected in various stains and over a half of the strains are found to have more than eight resistance genes, indicating that the proportion and quantity of ARGs are high in these strains and the drug resistance problem is serious. In this study, five amphenicols resistant genes were detected, of which, *floR* (70.2%, 33/47) and *cmlA* (53.2%, 25/47) genes are frequent, which are believed to be the main cause of amphenicol resistance in the strains. The detection rates of four aminoglycoside-modifying enzyme genes (*aac*(*3’*)*-IV, aadA1, aadA2, aac*(*3’*)*-IIc*) are higher than 50%, and multiple aminoglycoside modifying enzyme genes are detected in the same strains, resulting in high-level resistance to aminoglycosides. In this study, the detection rates of aminoglycosides resistance genes are basically consistent with the resistance phenotypes. However, five *aac*(*3*′)*-IV* positive strains are found susceptible to aminoglycosides, probably because of infective expression of the gene in these strains. *mcr-1i* is also frequently found in the strains. *mcr-1i* is present in plasmid and chromosome, but mainly plasmids. Plasmids could carry multiple ARGs (Zhu 2019). The high detection rate (40.4%) of *mcr-1* in this study likely results from overdose use of polymyxins in pig feed in Guizhou.

At present, the resistance of Gram-negative bacteria to β-lactam drugs is increasingly higher, and the most common resistance mechanism is that bacteria can produce three metalloenzymes: ESBL, cephalosporinase (AmpC enzyme) and carbapenems (Meini et al. 2019). The first two enzymes are the main causes for drug resistance of *E. coli* from livestock and poultry. This study detected five β-lactamase genes (the detection rate is 83.0%), including four ESBL genes (*bla*_TEM_, *bla*_CTX-M_, *bla*_SHV_, *bla*_OXA_) and one AmpC enzyme gene (*bla*_DHA_). There are 38 (80.9%) *E. coli* strains that produce ESBL, and these strains are more frequent to be resistant to various antimicrobials than the other nine strains that do not produce ESBLs. Extended-spectrum β-lactamase -positive strains can produce β-lactamase to hydrolyse β-lactamase antibiotics, it often leads to clinical failure of infection treatment. In the previous reports, CTX-M-type was most prevalent in ESBLs (Ho et al. 2011; Jiang, Kui & Huang 2015; Yang, Zeng & Lin 2019; Zeng 2016), but in this study, *bla*_TEM_ is the most popular in 38 ESBLs-positive strains (89.7%, 35/38), and it is basically consistent with the detection rate of 90.51% in Guizhou in 2016 (Cao et al. 2016), which should be taken seriously. *bla*_CTX-M-9G_ is most prevalent amongst CTX-Ms in this study (29.8%), conforming to the prevalence situation reported in this country (Jiang et al. 2015; Yang et al. 2019). Amongst 38 bacterial strains carrying *ESBLs* genes, eight complex *ESBLs* genotypes are detected, of which, the proportion of *bla*_TEM_+*bla*_CTX-M-9G_ is the highest (26.3%, 10/38). Furthermore, coexistence of *bla*_CTX-M-1G_ and *bla*_CTX-M-9G_ are found in ECP4 and ECP27, which carried four ESBLs genes. These findings showed that ESBLs genotypes are complex in the *E. coli* strains in Guizhou and would result in clinical challenges for infection control.

Low-resistant PMQR genes conferring quinolone resistances are shown to be closely related to the prevalence of ESBLs (Ma et al. 2009; Wu et al. 2007; Yang, Zhuang & Hua 2018), especially *qnr/oqxAB* and *bla*_CTX-M_ genes, which may be located on the same plasmid (Liu et al. 2014; Strahilevitz et al. 2009). In this study, PMQR genes were analysed in 17 *E. coli* strains and 14 strains were found to have *bla*_CTX-M_ with a detection rate of 82.4%, of which, *qnrS* and *oqxA* are detected at 58.8% and 52.9% of the strains. The complex genotype *bla*_CTX-M-9G_+*qnrS*+*oqxA* is detected in four strains. It is, therefore, likely that the ESBLs and PMQR genes are located on the same plasmid in the *E. coli* stains, which would somewhat increase the risk of horizontal transmission of ARGs. Previously, it was shown that *oqxA* and *oqxB* frequently coexist (Yang et al. 2018). However, this coexistence was not observed in our study, and further study is needed to clarify the situation.

## Conclusion

Extended-spectrum β-lactamase is widespread in *E. coli* from pigs in Guizhou and TEM-type is the most prevalent ESBLs. Most of the *E. coli* strains have multiple ESBLs. In CTX-M strains, CTX-M-9G is the most frequent; PMQR carriers are often detected in *bla*_CTX-M_-positive strains, and the two genes are likely located on the same plasmids. Therefore, the co-transmission needs to be addressed in the future. Only *oqxA* was detected but not *oqxB* in this study, indicating a need to clarify their relationship. Co-existence of *bla*_TEM_+*bla*_CTX-M-1G_+*bla*_CTX-M-9G_ in ECP4 and *bla*_TEM_+*bla*_CTX-M-1G_+*bla*_CTX-M-9G_+*bla*_SHV_ in ECP27 may allow further investigation of the position and position effect. In addition, over 40% strains are found to contain *mcr-1*, and the location of the gene needs to be defined.
